# Selection and validation of reference genes for qRT-PCR analysis of gene expression in *Microsporum canis* growing under different adhesion-inducing conditions

**DOI:** 10.1038/s41598-018-19680-9

**Published:** 2018-01-19

**Authors:** Anita Ciesielska, Paweł Stączek

**Affiliations:** Department of Microbial Genetics, Faculty of Biology and Environmental Protection, University of Łódź, Banacha 12/16, 90-237, Łódź, Poland

## Abstract

Dermatophytes are the group of filamentous fungi infecting keratinized structures such as skin, hair, and nails. Knowledge about genes and molecular mechanisms responsible for pathogenicity, as well as other biological properties of *Microsporum canis* is still relatively poor. The qRT-PCR is a reliable technique for quantifying gene expression across various biological processes, and choosing a set of suitable reference genes to normalize the expression data is a crucial step of this technique. We investigated the suitability of nine candidate reference genes: *β-act*, *β-tub*, *adp-rf*, *ef1-α*, *sdha*, *rpl2*, *mbp1*, *psm1*, and *rGTPa* for gene expression analysis in the dermatophyte *M. canis* in response to different carbon sources, phosphate levels, and pH shifts - factors that are extremely important and necessary for growth of dermatophyte in the host tissue. The transcription stability of these genes was evaluated using NormFinder, geNorm, BestKeeper, and RefFinder software. Regarding expression stability, *mbp1*, *β-act*, and *sdha* were the most stable housekeeping genes which we recommend for future qRT-PCR studies on *M. canis* strains. To the best of our knowledge this is the first study on selection and validation of reference genes for qRT-PCR data normalization in *M. canis* growth in culture media which promote adhesion-inducing conditions.

## Introduction

*Microsporum canis* is a member of dermatophytes – a group of pathogenic fungi able to invade keratinized structures, leading to infection of skin, hair, and nails. This species is distributed worldwide and in many areas such as Central and Southern Europe, Middle East, North Africa, South America, and China it is the most prevalent dermatophyte^1–11^ responsible for *tinea corporis* and *tinea capitis* in humans and animals^12^. The disease mainly affects preadolescents, the elderly, as well as immunocompromised individuals, including AIDS patients and transplant recipients^13–16^, and manifests by severe scalp itching, hair loss as well as skin scaling, especially around hair shafts^17,18^. Eradication of the disease is difficult because human asymptomatic carriers are common, moreover pets such as cats, dogs, and rabbits may transmit *M. canis* to humans^1,19–21^. Molecular mechanisms of interaction between dermatophytes and host cells are still poorly understood. Therefore, there is an explicit need for identification of factors involved in dermatophyte pathogenicity, and the logical starting point is identification of genes which expression increases or attenuates in response to different environmental stimuli typical for the stage of invasion. Analysis of expression profiles of such genes may enable to study more thoroughly virulence factors and mechanisms of resistance against common antifungal drugs. *M. canis* genome sequencing^22^ improved functional genomic studies to identify factors involved in dermatophyte pathogenicity. Dermatophyte adherence and secretion of enzymes are the key factors in colonization of the host tissues, which may be regulated in response to different carbon sources, phosphate levels, and ambient pH shifts^23^. Gene expression profiling is an effective means of studying response to different environmental stimuli and a necessary component in identifying genes and regulatory mechanisms associated with biological processes in any organism. Choosing a proper reference gene remains one of the golden rules to increase the credibility of qRT-PCR data interpretation^23^. Due to the limited knowledge on such genes suitable for expression analysis in dermatophytes^24^ we investigated the transcription level of a group of nine candidate housekeeping genes: *β-act* (β-actin), *β-tub* (β-tubulin), *adp-rf* (ADP ribosylation factor), *ef1-α* (elongation factor 1-alpha), *sdha* (succinate dehydrogenase complex flavoprotein subunit A), *rpl2* (ribosomal protein L2), *mbp1* (multiubiquitin chain binding protein 1), *psm1* (mitotic cohesion complex subunit Psm1), *rGTPa* (rho GTPase activating-protein 5) (Table 1) as reference genes for selected clinical strain of *M. canis* grown under different adhesion-inducing environmental stimuli typical for the stage of host infection, such as: different carbon sources (glucose, keratin, keratin with soy protein, elastin, collagen, colloidal chitin, keratinocyte free medium), low-Pi environments, pH shifts. The candidate reference genes were selected from among endogenous controls in some species of fungi as well as in other organisms^22,24^ and to the best of our knowledge this is the first such complex search in case of dermatophyte species. The stability of each candidate reference gene was evaluated by algorithms: geNorm module of qbase + (Biogazelle), NormFinder, BestKeeper, and RefFinder web-based comprehensive tool.

**Table 1:** *Microsporum canis* candidate reference genes used for qRT-PCR.

*Gene symbol*/accession no.	Gene name	Primers (5′-3′) forward reverse	Length bp	Tm [°C]	C_t_ range	Efficiency (%)	R^2^
***β-act*** XM_002845542	β-actin	CTCCTGAGGCTCTCTTCC GTAGTACCGCCGGACATG	142	60.5	15.08–18.70	110	0.99936
***β-tub*** XM_002848601	β-tubulin	AAGAGTTCCCAGACCGTATG TGTTGTACAAGGCCTCATTG	159	60.5	17.86–22.57	109	0.99975
***adp-rf*** XM_002848521	ADP ribosylation factor	GAATTCTCATGGTCGGTCTC AACGTTGAATCCGATGGTG	104	60.5	15.93–20.24	100	0.99855
***ef1-α*** XM_002850842	elongation factor 1-alpha	CCTAAGTTCGTCAAGTCTGG CTTCTCGACAGCCTTGATG	159	60.5	16.43–22.41	104	0.99798
***sdha*** XM_002843730	succinate dehydrogenase complex flavoprotein subunit A	TCTAGGAAACATGCACAAGG TTCGATAACACTCTGAGGGG	127	60.5	16.05–19.80	103	0.99895
***rpl2*** XM_002844911	ribosomal protein L2	GATCTATATTCACGGCTCGC ATGATGTTCTTCACGACACC	109	60.5	19.40–25.26	107	0.99912
***mbp1*** XM_002843632	multiubiquitin chain binding protein 1	AGTCCTAGTTACCTTGACCG CGGTGTTTAAGTGCTAGATAGG	123	60.5	18.73–22.18	99	0.99924
***psm1*** XM_002844171	mitotic cohesion complex subunit Psm1	AGCGTACCTGGATATTGAAG GGATAGCGAATAACAGAGCC	149	60.5	22.84–26.65	104	0.99980
***rGTPa*** XM_002843982	rho GTPase activating-protein 5	GACTCCCTCTGGCATATTTG ATCGGTTGCTTTCTCCTTC	160	60.5	16.44–18.97	104	0.99980

## Results

### Standard curve, PCR efficiency, and product specificity

**Figure 1:**
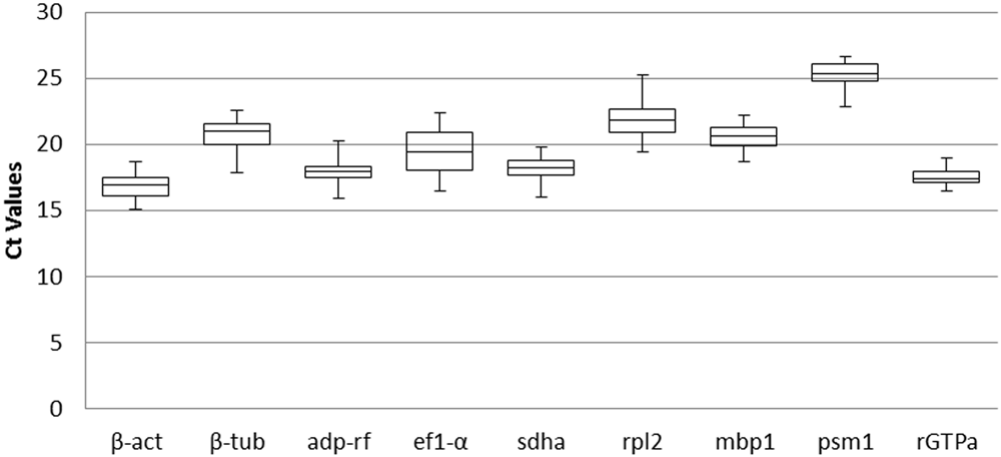
Expression level of nine reference genes in *M. canis*. The mean C_t_ values for all experimental conditions of each candidate reference gene are shown as box plot representations. The box indicates the 25^th^ and 75^th^ percentiles, the line across the box represents the median and whisker caps the maximum and minimum values.

**Figure 2:**
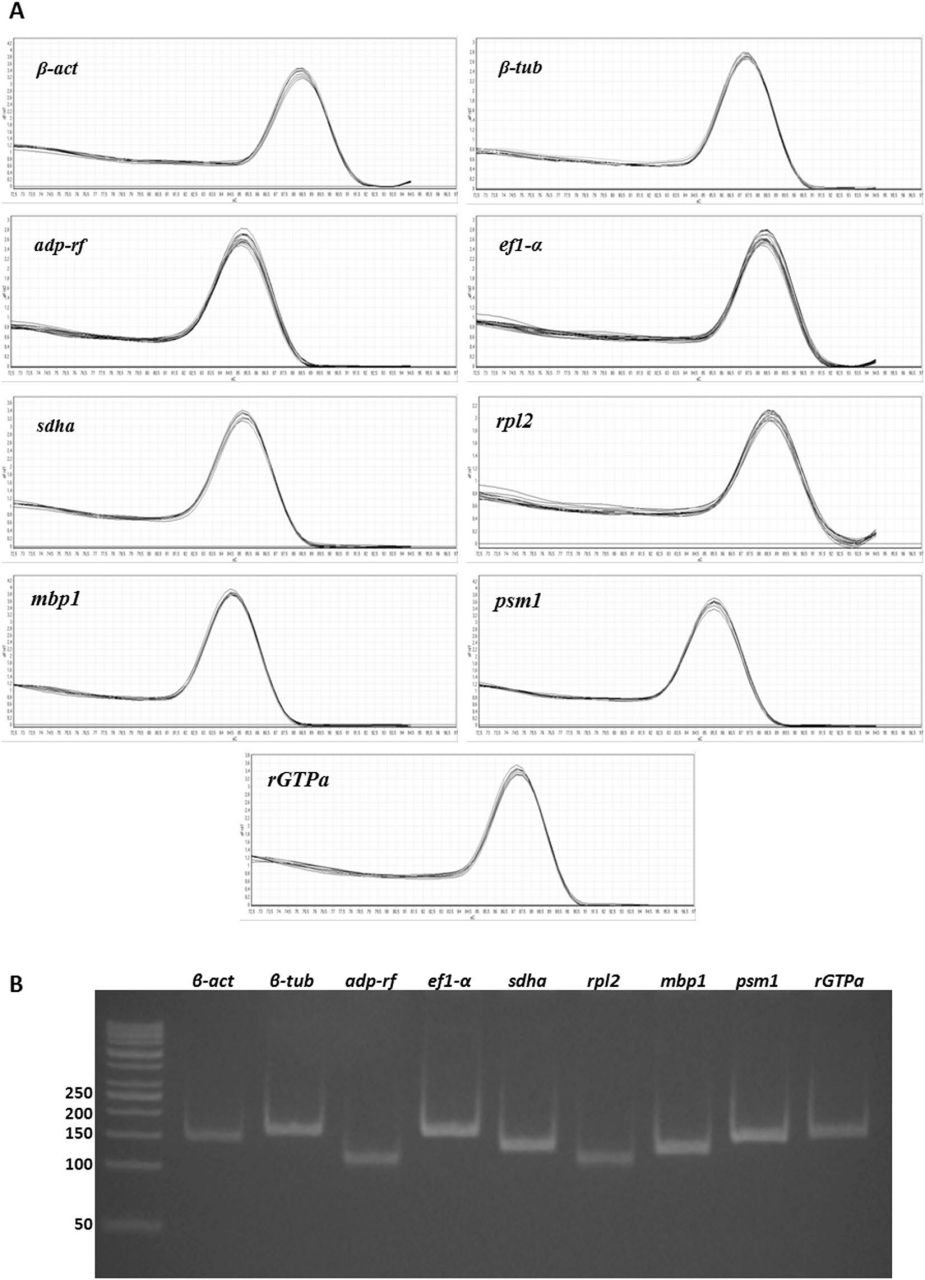
Melting curves of the nine *M. canis* candidate reference genes show single peaks (**A**). 8% polyacrylamide gel electrophoresis indicated the amplification of a single product of the expected size for nine reference genes (**B**).

The cDNA samples were obtained using total RNA isolated from three independent repetitions of *M. canis* cultures subjected to 30 environmental conditions (Supplementary Table S1). The expression of nine reference gene candidates (Table 1) was evaluated in control (*M. canis* cultivated in MM-Cove medium) and experimental conditions (Table S1). The raw C_t_ values (Supplementary Table S1) were used to calculate the mean C_t_ for each amplicon in each sample. The candidate reference genes exhibited C_t_ values ranging from 15.08–26.65 (Fig. 1, Table 1). The calculated efficiencies for the candidate reference genes, shown in Table 1, were between 99–110%. The efficiency curves for nine analyzed candidate genes were found to have linear correlation coefficients (R^2^) ranging from 0.997–0.999. Melt peak analysis demonstrated a single homogenous peak for all primer sets (Fig. 2A). Polyacrylamide gel electrophoresis analysis of the amplified products for all primer sets revealed single bands of the expected size (Table 1, Fig. 2B).

### Analysis of candidate reference gene expression using geNorm, NormFinder, BestKeeper, and RefFinder

**Figure 3:**
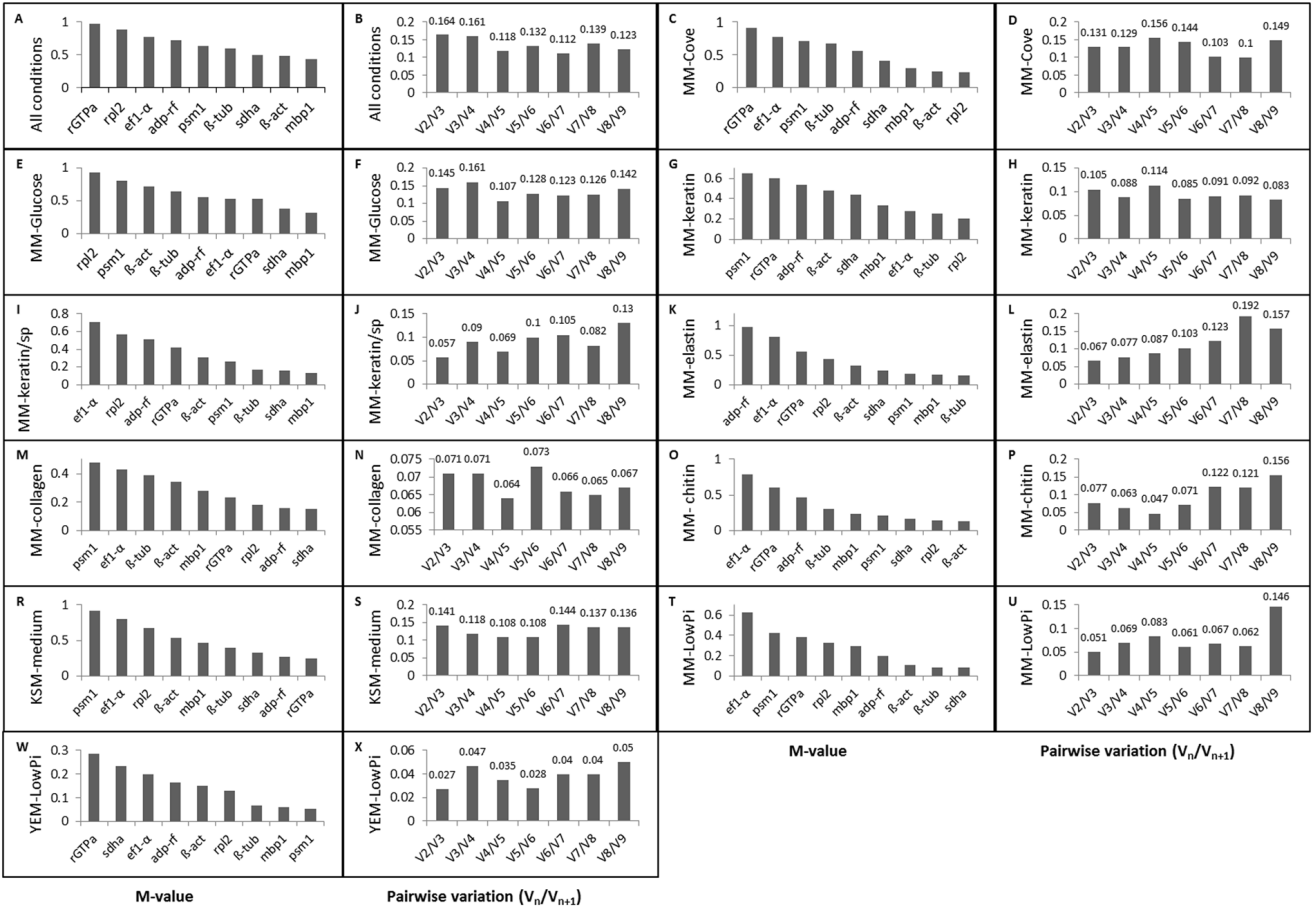
Gene expression stability (M-value) and pairwise variation (V_n_/V_n+1_) of the *Microsporum canis* candidate reference genes analyzed by geNorm.

The GeNorm module of qbase + (Biogazelle) ranks the reference genes based on the stability value (M-value). The lower M-value corresponds to the more stable gene. In order to examine the minimal number of genes required for reliable normalization, the V-value for all gene pairs was calculated (V_n_/V_n+1_) between two subsequent normalization factors (NF_n_ and NF_n+1_) with the cut-off value set at 0.15. Analyses were conducted using eleven groups of samples: A and B – all experimental conditions; C and D - growth in MM-Cove medium (control medium); E and F - growth in MM-Cove medium with glucose; G and H - growth in MM-Cove medium with keratin; I and J - growth in MM-Cove medium with keratin/soy protein; K and L - growth in MM-Cove medium with elastin; M and N - growth in MM-Cove medium with collagen; O and P - growth in MM-Cove medium with colloidal chitin; R and S - growth in KSM medium; T and U - growth in MM-LowPi medium; W and X - growth in YEM-LowPi medium. *mbp1*, *β-act*, *sdha*, and *β-tub* genes were the most stable genes under all tested conditions with *mbp1* ranked as the best reference gene (M-value = 0.438). On the other hand *rGTPa* was the least stable gene (M-value = 0.968) in all analyzed samples (Fig. 3A). The pairwise variation (V_n_/V_n+1_) indcated that the use of four reference genes was reliable for normalization (V_4/5_ = 0.118) (Fig. 3B). The stability analysis of nine candidate reference genes of *M. canis* cultivated on control medium (MM-Cove) showed that *rpl2* and *β-act* were the most stable ones (Fig. 3C), and the use of two reference genes was adequate to achieve credible data normalization (V_2/3_ = 0.131) (Fig. 3D). Given that *mbp1* and *sdha* genes presented the most stable expression level in the presence of glucose in a culture medium (Fig. 3E), we estimated according to pairwise variation (V_n_/V_n+1_) that the use of two reference gene gave reliable results for qRT-PCR data normalization (V_2/3_ = 0.145) (Fig. 3F). The stability analysis with geNorm module showed that *rpl2* and *β-tub* genes were the most stable when keratin was the main substrate during *M. canis* cultivation (Fig. 3G), and according to the pairwise variation (V_n_/V_n+1_) the use of these two reference genes was sufficient for the process of proper normalization (V_2/3_ = 0.105) (Fig. 3H). In the presence of keratin and soy protein in cultivation medium, *mbp1*, and *sdha* reference genes of *M. canis* reached the lowest M-value (Fig. 3I), and the use of two reference genes was suitable to achieve the best normalization data (V_2/3_ = 0.057) (Fig. 3J). The stability analysis of *M. canis* candidate reference genes expressed in MM-Cove medium supplemented with elastin showed that *β-tub* and *mbp1* genes were the most stable (Fig. 3K), while analysis of pairwise variation (V_n_/V_n+1_) revealed that the use of two reference genes was the best combination for normalization results (V_2/3_ = 0.067) (Fig. 3L). *sdha* and *adp-rf* were the most stable candidate reference genes of *M. canis* in the presence of collagen in a culture medium (Fig. 3M). Pairwise variation (V_n_/V_n+1_) demonstrated that the use of these two reference genes was reliable for accurate normalization (V_2/3_ = 0.071), moreover addition of one more gene (*rpl2*) resulted in variation of such normalization factor (V_3/4_ = 0.071) (Fig. 3N). β*-act* and *rpl2* reference genes were the most stable (Fig. 3O) when *M. canis* was cultivated on MM-Cove medium supplemented with colloidal chitin. According to (V_n_/V_n+1_) analysis the use of two genes was credible for normalization (V_2/3_ = 0.077) (Fig. 3P). *rGTPa* and *adp-rf* genes were ranked by geNorm as the best reference genes in Keratinocyte-SFM medium (KSM) containing L-glutamine (Fig. 3R), as the use of these genes resulted in accurate normalization (V_2/3_ = 0.141) (Fig. 3S). *Sdha* and *β-tub* were found to be the most stable genes in the presence of low concentration of organic Pi in MM-Cove medium at different pH (Fig. 3T) while *psm1* and *mbp1* were the most stable genes in the presence of low concentration of organic Pi in YEM- medium at different pH (Fig. 3W). In both cases, the use of two reference genes combination provided the best normalization (V_2/3_ = 0.051, and V_2/3_ = 0.027, respectively) (Figs. 3U, 3x). Summarizing, our analysis using geNorm module algorithms showed that *mbp1*, *β-act*, *sdha*, and *β-tub* with an M-value of 0.438, 0.478, 0.496, and 0.593, respectively (Table 2) were overall the most stable candidates for normalization of *M*. *canis* target gene expression across all experimental conditions. However, different sample sets required individual, most stable reference gene combinations (Fig. 3), what emphasizes the importance of such reference genes identification before qRT-PCR analysis.

**Table 2:** Ranking of *M. canis* candidate reference genes ordered by their expression stability, determined by geNorm, NormFinder, BestKeeper, and RefFinder for all experimental conditions.

Rank	geNorm		NormFinder		BestKeeper		RefFinder	
	Gene	Expression Stability (M)	Gene	Stability Value (p)	Gene	Coefficient of Correlation (r)	Gene	Geomean of ranking values
**1**	***mbp1***	**0.438**	***sdha***	**0.081**	***β-act***	**0.898**	***mbp1***	**1.178**
**2**	***β-act***	**0.478**	***mbp1***	**0.102**	***mbp1***	**0.891**	***sdha***	**2.060**
**3**	***sdha***	**0.496**	***β-act***	**0.184**	***sdha***	**0.876**	***β-act***	**2.449**
4	*β-tub*	0.593	*β-tub*	0.244	*β-tub*	0.821	*rGTPa*	3.807
5	*psm1*	0.636	*psm1*	0.327	*ef1-α*	0.802	*adp-rf*	4.162
6	*adp-rf*	0.721	*adp-rf*	0.334	*psm1*	0.761	*psm1*	4.427
7	*ef1-α*	0.774	*ef1-α*	0.381	*adp-rf*	0.716	*β-tub*	6.735
8	*rpl2*	0.886	*rpl2*	0.413	*rpl2*	0.691	*ef1-α*	8.239
9	*rGTPa*	0.968	*rGTPa*	0.464	*rGTPa*	0.492	*rpl2*	8.739

NormFinder analysis showed that *sdha* had the lowest stability value SV = 0.081 and demonstrated that when the samples were subjected to different environmental stimuli the best combination of two reference genes was *sdha* and *mbp1* with SV = 0.070 (Table 3). NormFinder demonstrated that *mbp1* was the most stable gene during *M. canis* growth in MM-Cove medium supplemented, respectively, with glucose (SV = 0.053), keratin (SV = 0.063), keratin with soy protein (SV = 0.188), elastin (SV = 0.040), collagen (SV = 0.096), and colloidal chitin (SV = 0.106). *sdha* was the most stable gene during *M*. *canis* growth in MM-Cove medium (SV = 0.096), KSM medium (SV = 0.224), and MM-Cove medium with low concentration of organic Pi at different pH shifts (SV = 0.093). In case of *M*. *canis* growth in YEM medium with low concentration of organic Pi and at different pH, NormFinder analysis demonstrated that *β-tub* was the most stable reference gene (SV = 0.254).

**Table 3:** Ranking of *M. canis* candidate reference genes determined by NormFinder algorithm.

Rank	All conditions		MM-Cove growth		Glucose growth		Keratin growth		Keratin/soy protein growth		Elastin growth		Collagen growth		Colloidal chitin growth		KSM Medium growth		MM-LowPi medium growth		YEM-LowPi medium growth	
	gene	SV	gene	SV	gene	SV	gene	SV	gene	SV	gene	SV	gene	SV	gene	SV	gene	SV	gene	SV	gene	SV
1	*sdha*	0.081	*sdha*	0.096	*mbp1*	0.053	*mbp1*	0.063	*mbp1*	0.188	*mbp1*	0.040	*mbp1*	0.096	*mbp1*	0.106	*sdha*	0.224	*sdha*	0.093	*β-tub*	0.254
2	*mbp1*	0.102	*mbp1*	0.096	*sdha*	0.057	*sdha*	0.117	*β-act*	0.250	*sdha*	0.100	*sdha*	0.113	*β-act*	0.280	*mbp1*	0.269	*β-act*	0.182	*psm1*	0.319
3	*β-act*	0.184	*rpl2*	0.323	*ef1-α*	0.207	*rpl2*	0.173	*sdha*	0.272	*β-act*	0.141	*β-act*	0.230	*sdha*	0.315	*β-act*	0.359	*β-tub*	0.212	*ef1-α*	0.325
4	*β-tub*	0.244	*β-act*	0.345	*adp-rf*	0.248	*adp-rf*	0.210	*β-tub*	0.414	*β-tub*	0.175	*β-tub*	0.252	*rpl2*	0.334	*ef1-α*	0.553	*mbp1*	0.248	*mbp1*	0.326
5	*psm1*	0.327	*adp-rf*	0.420	*β-act*	0.252	*β-act*	0.220	*adp-rf*	0.440	*psm1*	0.176	*ef1-α*	0.310	*adp-rf*	0.363	*adp-rf*	0.557	*rpl2*	0.299	*sdha*	0.337
6	*adp-rf*	0.334	*psm1*	0.543	*β-tub*	0.303	*β-tub*	0.235	*rGTPa*	0.567	*rpl2*	0.254	*psm1*	0.314	*psm1*	0.427	*β-tub*	0.610	*psm1*	0.369	*rpl2*	0.428
7	*ef1-α*	0.381	*β-tub*	0.562	*rpl2*	0.305	*ef1-α*	0.277	*psm1*	0.593	*adp-rf*	0.400	*rpl2*	0.324	*β-tub*	0.468	*psm1*	0.645	*adp-rf*	0.375	*β-act*	0.431
8	*rpl2*	0.413	*ef1-α*	0.587	*psm1*	0.377	*psm1*	0.302	*ef1-α*	0.664	*ef1-α*	0.448	*adp-rf*	0.337	*rGTPa*	0.542	*rGTPa*	0.912	*rGTPa*	0.492	*adp-rf*	0.437
9	*rGTPa*	0.464	*rGTPa*	0.921	*rGTPa*	0.392	*rGTPa*	0.390	*rpl2*	0.767	*rGTPa*	0.474	*rGTPa*	0.374	*ef1-α*	0.737	*rpl2*	1.151	*ef1-α*	0.547	*rGTPa*	0.583
best combination	*sdha, mbp1*	0.070	*a*	—	*a*	—	*a*	—	*a*	—	*a*	—	*a*	—	*a*	—	*a*	—	*a*	—	*a*	—

The Excel-based BestKeeper algorithm^25^ was used to evaluate the expression stability of reference genes based on the coefficient of variance (CV) and the standard deviation (SD) of the average C_t_ values. The descriptive statistics for the nine candidate genes is given in Table 4. Among nine evaluated candidates, seven genes exhibited a recommended standard deviation value [0.5 < SD[±C_t_] ≤ 1.00]. Data analysis using pairwise correlation and regression analysis assessed the inter-gene relations, which eliminated *ef1-α* and *rpl2* genes as showing highest standard deviation (SD = 1.21, and 1.04, respectively) and variation (CV = 6.25, and 4.75, respectively). The lowest variation was observed for the *rGTPa* gene (CV = 2.74). Moreover, *rGTPa* gene demonstrated a weak correlation to BestKeeper index (BI) as compared to other candidates (r = 0.492). Therefore, *ef1-α*, *rpl2*, and *rGTPa* gene were excluded from further normalization. The analysis of the remaining six genes (*β-act*, *β-tub*, *adp-rf*, *mbp1*, and *psm1*) showed a significant correlation of 0.716 < r < 0.898 between their expression levels and BI (p < 0.001). Ranking of the four tightly correlated genes on the basis of variation from the most stable to the least stable one was as follows: *β-act*, *mbp1*, *sdha*, and *β-tub*. Among these four genes *β-act*, and *mbp1* were the most highly correlated 0.898 < r < 0.891 to the BI (Table 4).

**Table 4:** Descriptive statistical analysis of candidate reference genes using BestKeeper.

Gene	*β-act*	*β-tub*	*adp-rf*	*ef1-α*	*Sdha*	*rpl2*	*mbp1*	*psm1*	*rGTPa*
n	90	90	90	90	90	90	90	90	90
GM [C_t_]	16.73	20.75	17.86	19.37	18.15	21.79	20.58	25.25	17.48
AM [C_t_]	16.75	20.78	17.88	19.43	18.17	21.83	20.60	25.27	17.49
Min [C_t_]	15.08	18.87	15.93	16.43	16.05	19.41	18.74	22.84	16.44
Max [C_t_]	18.70	22.57	20.25	22.41	19.81	25.27	22.19	26.66	18.98
SD [±C_t_]	**0.72**	**0.83**	**0.60**	1.21	**0.70**	1.04	**0.66**	**0.71**	**0.48**
CV [% C_t_]	4.30	3.99	3.37	6.25	3.83	4.75	3.22	2.79	2.74
Min [x-fold]	−3.12	−7.39	−3,79	−7.66	−4.28	−5.20	−3.60	−5.31	−2.06
Max [x-fold]	3.94	3.53	5.26	8.23	3.16	11.17	3.05	2.65	2.82
SD [±x-fold]	1.65	1.78	1.52	2.32	1.62	2.05	1.58	1.63	1.39
BI Index [r]	**0.898**	**0.821**	**0.716**	0.802	**0.876**	0.691	**0.891**	**0.761**	0.492
p-value	0.001	0.001	0.001	0.001	0.001	0.001	0.001	0.001	0.006

Finally, RefFinder which is a comprehensive web-based tool that integrates geNorm, NormFinder and BestKeeper was applied to generate a comprehensive final ranking of the candidate reference genes. As shown in Table 2, *mbp1*, *sdha*, and *β-act* were ranked as the top stable genes under all experimental conditions.

### Stability of *mbp1*, *β-act* and *sdha* reference genes in *M. canis* strains

**Figure 4:**
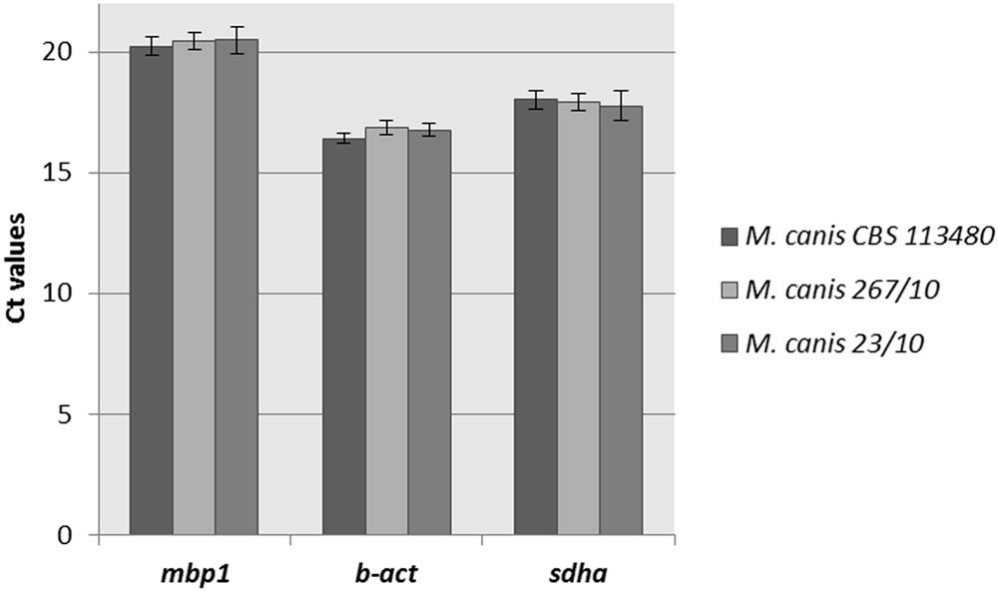
Stability of *mbp1*, *β-act* and *sdha* reference genes expression, evaluated in three *M. canis* strains cultured on MM-Cove medium (control medium) and MM-Cove medium supplemented with keratin. Gene expression levels are represented by average C_t_ values. *mbp1* (*p* = 0.91, ANOVA), *β-act* (*p* = 0.46, ANOVA) and *sdha* (*p* = 0.93, ANOVA) gene expression level was not significantly different across analyzed culture conditions. Error bars indicate standard error.

We compared the data of *mbp1*, *β-act*, and *sdha* reference genes expression in *M*. *canis* CBS 113480 and *M*. *canis* 23/10 strain (isolated from cat) with those obtained for *M. canis* 267/10 strain, which was used in the stability evaluation. The *M. canis* CBS, *M*. *canis* 23/10, and *M*. *canis* 267/10 strain were cultivated at 28 °C for 48 h in MM-Cove medium (control medium), and MM-Cove medium supplemented with keratin. Degradation of keratin as well as other proteins releases amino acids, metabolism of which leads to secretion of ammonia what changes pH from acidic to alkaline, after 48 hours^22^, which is extremely important for growth of dermatophytes in the host tissue. Therefore, to check stability of the selected reference genes in other *M. canis* strains it is reasonable to choose keratin as a carbon source, as it builds *stratum corneum* of mammalia. We observed slight variation in the C_t_ values (Fig. 4) but *mbp1*, *β-act*, and *sdha* genes expression was not significantly different under the experimental conditions (*p*_mbp1_ = 0.91; *p*_β-act_ = 0.46; *p*_sdha_ = 0.93, ANOVA) (Fig. 4). These results confirmed the stability of *mbp1*, *β-act*, and *sdha* reference gene expression in different *M. canis* strains, suggesting that these genes are sufficient for effective normalization of qRT-PCR data.

### Validation of selected *M. canis* reference genes

**Figure 5:**
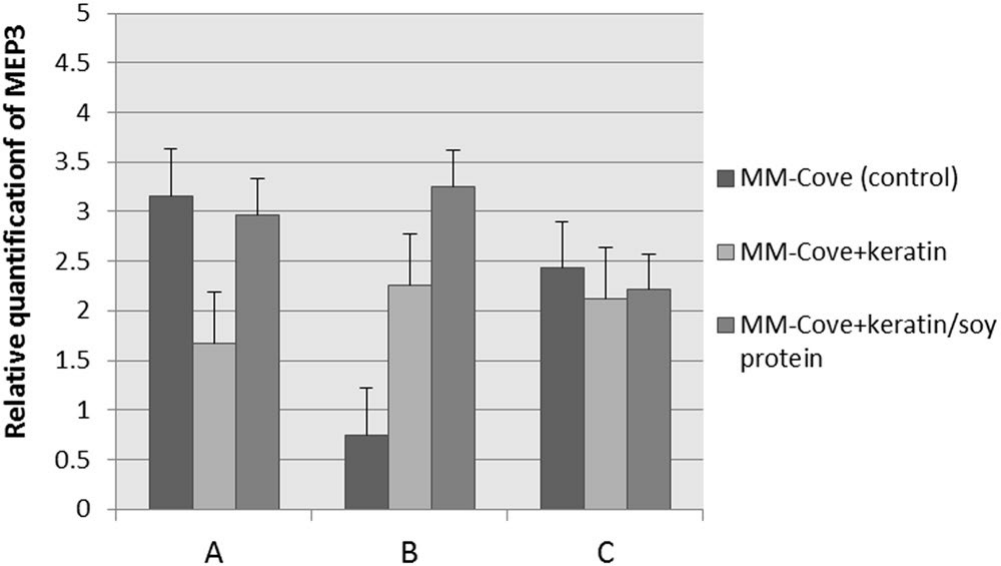
Relative quantification of *MEP3* gene expression in control, keratin and keratin/soy protein samples using different reference gene variants: A (*p* = 0.67, ANOVA) - two least stable reference genes *rpl2* and *rGTP*; B (*p* = 0.72, ANOVA) – three most stable reference genes *mbp1*, *β-act* and *sdha*; C (*p* = 0.55, ANOVA) – all candidate reference genes. Error bars indicate standard error.

To validate the selected reference genes for qRT-PCR normalization, the relative expression profiles of the *MEP3* gene were analyzed. *MEP3* is known as the gene encoding *M. canis* keratynolityc metalloprotease (43.5 kDa) expressed in the presence of keratin^26^. The validation analysis was conducted using three different reference gene variants selected by GeNorm, NormFinder, BestKeeper, and RefFinder in *M. canis* 267/10 strain cultivated at 28 °C for 48 h in MM-Cove medium (control medium), MM-Cove medium supplemented with keratin, and MM-Cove medium supplemented with keratin and soy protein which increases proteolytic activity. These variants were as follows: variant A - two least stable reference genes (*rpl2* and *rGTP*); variant B - three most stable reference genes (*mbp1*, *β-act*, *sdha*); variant C - all candidate reference genes. Relative expression was calculated using the 2^−ΔΔCt^ method. The statistical significance between control and treatment conditions was analysed by one-way ANOVA test. The best result indicating an increase of *MEP3* transcript of *M. canis* 267/10 growing in MM-Cove medium supplemented with keratin and keratin/soy protein in relation to control medium was found when the three reference genes (variant B) were used, as revealed by all four algorithms (Fig. 5).

## Discussion

qRT-PCR technique is a frequently used platform to quantify transcript abundance. However its heavily dependent on the stability of the internal reference genes used to normalize measurements of transcription level. Normalizer genes are defined as those with a stable expression under previously defined conditions, thus appropriate to quantify gene expression level of specific targets. In agreement with our results, increasing numbers of studies demonstrated that no single reference gene is stably expressed in all cell types and under different experimental conditions^27^. Therefore, the expression stability of candidate reference genes needs to be verified before each qRT-PCR experiment.

In the present study, we used four different algoritmhs such as geNorm, NormFinder, BestKeeper, and RefFinder to evaluate nine candidate reference genes for their potential use as internal controls for expression analysis of target genes of *M. canis* in culture media supplemented with different carbon sources, inorganic phosphate, and at optimal pH what promotes adhesion-inducing conditions. To our knowledge, this is the first identification and validation of *mbp1*, *β-act*, and *sdha* as the most suitable and stably expressed reference genes of *M*. *canis* tested among the larger set consisting of nine candidates (Table 2). Transcription factor 1 (*mbp1*) involved in transition of cell cycle from G1 to S phase was classified in our study as one of the three most stable genes (Table 2). This is the unique use of this gene as there have been no reports on validation of *mpb1* as a reference in other fungi transcription studies so far.

*β-act*, one of the three major proteins of cytoskeleton and *sdha* gene that codes for a subunit of succinate dehydrogenase and is important in cellular respiration were also found in our study to be suitable reference genes for expression analyses in *M*. *canis* growing under different adhesion inducing conditions. *β-act* as well as *18 S* and *gapdh* were some of the most commonly used housekeeping genes in qRT-PCR analysis^28^. *β-act*, performed as highly reliable reference gene when evaluated in fungi such as *Trichophyton rubrum*^24^, *Aspergillus niger*^29^, *Candida albicans*^30^ but also in other eukariots such as: barley (*Hordeum vulgare* L.)^31^, tung tree (*Vernicia fordii* Hemsl.)^32^, crop *Musa*^33^ or humans (prostate cancer studies)^34^. However, it has to be pointed out that there were also reports in which *β-act* was found to be unstable e.g. in *Saccharomyces cerevisiae*^35^, *Candida glabrata*^36^, *Benjaminiella poitrasii*^37^, *Poria cocos*^38^, *Monopteus albus*^39^ or cell culture models infected with influenza viruses^40^. *Sdha* gene was found to be suitable housekeeping gene for expression analyses in bovine tuberculosis^41^, rat tissue under toxicological conditions^42^, human glioma^43^, stress response of the athlete horses cells^44^, neutrophils, and untreated total blood leukocytes^45^. Again, some other studies have demonstrated that the stability of *sdha* gene expression was low in *Candida glabrata*^36^, rat vocal fold model of mucosal injury^46^ or different tissues of yak fetuses^47^. So, despite the fact that *β-act* and *sdha* genes were often used as housekeeping genes, a number of studies have provided evidence that their transcription level may vary between different organisms, cell types, developmental stages or experimental conditions^27^ and for that reason the selection of the most appropriate internal controls for each experimental model used for qRT-PCR is a crucial prerequisite for reliable gene expression analysis. In this study several optimal sets of reference genes that are suitable for qRT-PCR data normalization in *M. canis* were identified (Table 3, Fig. 3). These results indicate that the stability of reference gene expression in *M. canis* needs to be investigated for each experimental condition, what confirms the thesis that there is no universal reference gene (Fig. 3, Table 3).

In order to validate the reliability of the selected reference genes, relative quantification of *MEP3* gene known to be induced in the presence of keratin was performed. The *MEP3* up-regulation was detectable in *M. canis* 267/10 growing in MM-Cove medium supplemented with keratin and also with keratin/soy protein only when the three reference genes: *mbp1*, *β-act*, and *sdha* previously selected as most stable internal controls were used in combination. The results clearly proved that normalization using inappropriately chosen housekeeping genes could lead to erroneous conclusions (Fig. 5).

In conclusion, our study was the first attempt to evaluate and validate *M. canis* reference genes. These findings will allow further analysis of *M. canis* gene expression under different adhesion-inducing environmental stimuli, with improved accuracy and reliability, and may also provide a starting point for selection of candidate genes for gene expression analysis in other related species.

## Materials and Methods

### Selection of candidate genes and primer design

Nine candidate reference genes (*β-act*, *β-tub*, *adp-rf*, *ef1-α*, *sdha*, *rpl2*, *mbp1*, *psm1*, and *rGTPa*) were selected based on the NCBI database (http://www.ncbi.nlm.nih.gov) in some fungal species and other organisms (Table 1). Primers were designed based on nucleotide sequences deposited in GenBank (Table 1) and theoretically evaluated using Primer3 software^48^. Default program settings were selected except for following categories: “monovalent cations concentration” which was set at 50 mM, “divalent cations concentration” set at 3 mM, “dNTP concentration” set at 1.2 mM, and “annealing oligo concentration” set at 250 mM. All PCR products were within 80–150 bp range and one of the primers from each pair was anchored within exon-exon junction. Each primer pair underwent experimental evaluation and was accepted if all following conditions were true: (1) product PCR reaction using cDNA as a template was specific, (2) reaction using genomic DNA as a template gave no product, and (3) the efficiency of a real time PCR reaction was between 90–110% (Table 1).

### Fungal strain and growth conditions

For all experiments *Microsporum canis* 267/10 strain was used, which is a clinical isolate from *tinea capitis* of a 54 years old woman. *Microsporum canis* CBS 11348 and *Microsporum canis* 23/10, a clinical isolate from cat, were used in evaluation of the most stable reference genes, selected by geNorm, NormFinder, BestKeeper, and RefFinder algorithms. Standard mycological identification based on the phenotypic features was performed in the specialized hospital laboratory and confirmed in our laboratory by PCR-RFLP analysis targeting ITS1–5.8S-ITS2 followed by sequencing^49^. Conidia from *M. canis* strain (approximately 10^7^ cells/ml) were isolated as described previously^50^ and incubated separately for 24 h, 48 h, and 72 h at 28 °C with agitation in a liquid minimal medium (MM-Cove)^51^ (control medium) supplemented with 70 mM sodium nitrate at pH 5.0, and, respectively, with different carbon sources such as: glucose (55 mM), 0.5% (w/v) keratin, 0.5%/1% (w/v) keratin/soy protein, 0.25% (w/v) elastin, 0.25% (w/v) collagen, or 1% (w/v) colloidal chitin, as well as in Keratinocyte serum- free medium (1X) (Thermo Scientific). The *M. canis* conidia were also inoculated into Low-Pi minimal liquid medium (MM), and yeast extract medium (YEM) pH 5.0, 8.0, 10.0 and incubated at 37 °C for 17 h at 200 rpm. Final concentrations of Pi in low-Pi cultures was 200 μmol in case of MM-medium and 700 μmol in YEM medium^52^.

### RNA extraction, cDNA synthesis and qRT-PCR

Total RNA was extracted from *M*. *canis* cells using RNeasy Plant Mini Kit (Qiagen) according to the manufacturer’s instructions, with addition of DNase I to eliminate potential DNA contamination. Quantity and purity of the RNA was assessed by NanoPhotometer^TM^ Pearl Version 1.0 (IMPLEN). Only RNA samples with A260/A280 ratio between 1.9 and 2.1, and A260/A230 ratio higher than 2.0 were used in the analysis. RNA integrity was further assessed by 1% denaturing agarose gel electrophoresis. Two micrograms of total RNA were reverse transcribed into cDNA to a final volume of 40 µl using RevertAid Transcriptase (Thermo Scientific) according to the manufacturer’s instructions. qRT-PCR was performed in RotorGene Q System (Qiagen). The reaction mixture (20 µl) contained 10 µl of SsoAdvanced Universal SYBR®Green Supermix (2X), 1 μl of each primer (500 nM), 5 μl of diluted cDNA (1:40) and 4 μl of nuclease-free water. Amplifications were performed using the following cycling profile: an initial activation step (95 °C for 1 min) followed by 40 cycles of denaturation at 95 °C for 20 s, annealing at 60.5 °C for 20 s, and extension at 72 °C for 15 s. For melting curve analysis, a dissociation step cycle (72 °C for 10 s, and then 0.5 °C for 10 s until 95 °C) was added. All qRT-PCR experiments were performed in three biological and three technical replicas. Amplification efficiency (E) and correlation coefficient (R^2^) were calculated using the Rotor-Gene Q Series Software Version 2.3.1. (Qiagen) by standard curve method with 4-fold serial dilutions (Table 1).

### Data analysis

The expression stability of the 9 reference genes was evaluated by geNorm module of qbase + Version 3.1 (Biogazelle), NormFinder, BestKeeper and RefFinder algorithms in 90 samples (three biological replicas and 30 different conditions, Supplementary Table S1) under different experimental conditions. The geNorm module was used to compute expression stability values for all reference targets. As an input for analysis, C_t_ values exported directly from the Rotor-Gene Q Series Software Version 2.3.1. (Qiagen) were used. The candidate reference genes were ranked according to the expression stability M value, which is the average pairwise variation of a particular gene with all other reference genes. The gene showing the lower M-value is assigned to be expressed in a more stable fashion, while the one with the higher M-value has the less stable expression^53^. This algorithm was used to rank the optimal number of reference genes for each experimental condition. The geNorm module determines the pairwise variation V_n_/V_n+1_ between two subsequent normalization factors NF_n_ and NF_n+1_ in order to rank the minimum number of reference genes for normalization with a cut-off value of 0.15. NormFinder is a Visual Basic application software which takes into account intra- and inter-group variations and calculates a stability M-value. The genes with lower M-values indicate low inter- and intra-group variations and are considered to have greater stability^54^. The BestKeeper algorithm is usually performed by assessing the calculation of standard deviation SD (±C_t_) and correlation coefficients of variance CV (%C_t_) for all reference genes in all samples. All stably expressed reference genes are combined into the BestKeeper index using the geometric mean of each candidate reference gene’s C_t_ value^25^. The webbased tool RefFinder (http://leonxie.esy.es/RefFinder/) was used in order to combine the results and rank the candidate genes. The lowest rank indicates the most stably expressed gene.

## Electronic supplementary material

Supplementary Information
