# Supplementary material for: Selection and validation of reference genes for qRT-PCR analysis of gene expression in Microsporum canis growing under different adhesion-inducing conditions
Source: Sci Rep. 2018 Jan 19;8:1197. doi: 10.1038/s41598-018-19680-9 (PMC5775245; doi:10.1038/s41598-018-19680-9)
Supplement: Supplementary file 1 — Supplementary Information [file 41598_2018_19680_MOESM1_ESM.pdf]

SUPPLEMENTARY INFORMATIONS

Selection and validation of reference genes for qRT-PCR analysis of gene expression in *Microsporum canis* growing under different adhesion-inducing conditions

Anita Ciesielska*, Paweł Stączek

Department of Microbial Genetics, Faculty of Biology and Environmental Protection, University of Łódź, Banacha 12/16, 90-237 Łódź, Poland

*Corresponding author: anita.ciesielska@biol.uni.lodz.pl

TABLE

Supplementary Table S1. Averaged raw C_t values of the *M. canis* samples in different experimental conditions.

Table S1. Averaged raw C_t values of the *M. canis* samples in different experimental conditions.

**Experimental conditions**	***β-act***	***β-tub***	***adp-rf***	***ef1-α***	***sdha***	***rpl2***	***mbp1***	***psm1***	***rGTPa***
**C**-24h,28°C	15,0816	17,8663	16,0168	16,4336	16,0507	19,4089	18,7353	22,8421	17,4922
**C**-48h,28°C	16,7397	21,1253	18,2989	19,8562	18,1715	20,9306	20,4721	26,2977	17,3464
**C**-72h,28°C	15,3093	20,5259	18,3139	17,5091	17,5893	19,7025	19,7365	25,2794	16,9789
**G**-24h,28°C	15,6827	18,8063	16,4067	17,4261	16,5092	20,8353	19,2533	23,1385	16,6919
**G**-48h,28°C	17,6641	21,5370	17,3173	19,1357	18,4533	20,2351	20,6511	26,6561	17,1660
**G**-72h,28°C	15,4841	20,8007	17,7020	17,7954	17,8440	19,9515	19,8217	25,4514	17,2258
**Ker**-24h,28°C	15,9188	19,9406	17,2388	17,8755	17,3716	20,1980	19,7845	24,8051	17,1868
**Ker**-48h,28°C	16,7456	22,0708	17,9580	19,8456	18,1545	22,2683	21,2166	26,5231	18,2382
**Ker**-72h,28°C	18,7026	22,5715	18,7174	21,1279	19,8088	23,1328	22,1256	26,0966	18,3195
**KS-**24h,28°C	17,0917	20,9406	18,2715	19,1324	18,6174	22,8633	21,0194	24,8273	18,1798
**KS-**48h,28°C	16,2997	19,4097	17,8953	20,4286	16,8764	22,8517	19,5165	23,7266	17,0533
**KS-**72h,28°C	17,8767	21,5299	18,1868	20,8875	18,7592	23,2783	21,3891	25,8963	17,8543
**Col-**24h,28°C	16,1507	19,7351	17,2014	18,0610	17,8255	21,6905	19,9646	24,3561	17,4721
**Col-**48h,28°C	15,2868	19,5621	15,9342	17,9221	16,6321	20,1406	19,2660	24,6989	16,6312
**Col-**72h,28°C	17,5518	21,0597	17,5074	19,8285	18,3310	22,1783	20,9918	25,6950	17,9076
**E-**24h,28°C	16,4378	20,4787	18,0605	18,1672	18,4145	22,0419	20,6815	25,3157	18,6170
**E-**48h,28°C	17,0030	20,3940	20,2496	20,9746	18,0428	21,5316	20,6362	25,6160	17,2521
**E-**72h,28°C	17,7627	21,1027	18,1437	20,6481	18,4554	23,3477	21,1107	26,1691	17,7786
**CH-**24h,28°C	16,9151	20,1510	18,6663	22,4127	17,5768	21,4116	20,7589	25,0624	18,9790
**CH-**48h,28°C	16,9125	20,0182	18,9066	21,1125	17,9020	21,3655	20,5758	25,4995	17,3631
**CH-**72h,28°C	17,2627	20,9555	17,5924	19,5317	17,8596	21,8061	20,6294	25,2594	17,4488
**5Y-**17h, 37°C	17,1928	21,2574	17,9310	19,2751	18,7825	22,1538	21,6058	25,4353	16,8214
**8Y-**17h,37°C	17,6927	21,7832	18,1159	19,3595	19,7826	22,4862	22,1890	26,0458	18,1632
**10Y-**17h,37°C	17,4187	21,0490	17,8458	19,4211	19,2702	22,1799	21,5811	25,3638	17,5222
**5M-**17h,37°C	16,3326	20,0034	17,1083	18,1658	17,7268	21,0094	20,8181	24,8594	17,4035
**8M-**17h,37°C	17,0149	20,9796	18,16168	21,0196	18,6791	21,9383	21,3397	25,0264	17,3173
**10M-**17h,37°C	17,6266	21,5597	18,19993	21,5997	19,2398	21,6265	21,3499	26,3299	18,0593
**KSM-**24h,28°C	17,0920	22,0952	17,66209	20,8926	18,7673	24,3728	20,6387	26,4526	16,4423
**KSM-**48h,28°C	16,4392	22,0064	18,82971	19,0363	18,9923	25,2676	20,1296	24,7893	17,3724
**KSM-**72h,28°C	15,7516	22,0205	17,82068	17,8888	18,6402	22,5988	19,9818	24,4985	16,5025

C-MM-Cove medium (control medium); **G**-MM-Cove+glucose; **Ker**-MM-Cove+keratin; **KS**-MM-Cove+keratin/soy protein; **Col**-MM-Cove+collagen; **E**- MM-Cove+elastin; **CH**-MM-Cove+colloidal chitin; **5Y**-YEM Low Pi medium pH 5.0; **8Y**- YEM Low Pi medium pH 8.0; **10Y**- YEM Low Pi medium pH 10.0; **5M**-MM-Cove LowPi medium pH 5.0; **8M**- MM-Cove LowPi medium pH 8.0; **10M**- MM-Cove LowPi medium pH 10.0, **KSM**-Keratinocyte-SFM medium.
